# Classification of interval breast cancers and their presentation since screening: impact on long-term survival

**DOI:** 10.1186/bcr3302

**Published:** 2012-11-09

**Authors:** YF Fong, J Evans, D Brookes, K Gower Thomas

**Affiliations:** 1 Royal Glamorgan Hospital, Llantrisant, UK; 2 Breast Test Wales, Cardiff, UK

## Introduction

Interval cancers (IC) present between screenings. All interval cancers within Breast Test Wales (BTW) are classified into true interval (TI), false negative (FN), occult (OCC) and unclassified (UCC). We aim to evaluate the overall number of IC within BTW and their presentation since screening and the impact on long-term survival.

## Methods

All women with IC having had screening between 1998 and 2001 were included. Classifications of IC were performed during the IC review process. Date of screening and date of diagnosis were used to calculate the interval between screening and diagnosis. Ten-year survival data were obtained on all interval cancers from national register.

## Results

Some 692 interval cancers were diagnosed following screening. In total, 57.8% (391) were TI, 17.7% (120) were FN, 10% (68) were OCC and 14.5% were UCC. A total of 15.6% (108) presented within 1 year of screening, 38.2% (264) in the second year, and 46.1% (319) in the third year since screening. The overall 10-year survival was 72.4%. It was 77.5% for TI and 55% for FN. The odds ratios for cancer presenting in the second year and third year were 1.009 (*P *= 0.97) and 1.1162 (*P *= 0.486), respectively, as compared with IC presenting within the first year.

## Conclusion

The majority of IC were TI and presented in the third year since screening. FN had worse 10-year survival compared with TI. IC presenting in the third year did not have a statistically significantly worse long-term survival. Reduction of the screening interval would not have an impact on long-term survival outcome.

